# Cross-sectional Analysis of Dermatologists and Sponsored Content on TikTok

**DOI:** 10.2196/44413

**Published:** 2023-04-21

**Authors:** Denisse Cristina Porras Fimbres, Alyssa P Quinn, Benjamin R Cooper, Colby L Presley, Jennifer Jacobs, Chandler W Rundle, Robert P Dellavalle

**Affiliations:** 1 School of Medicine Duke University Durham, NC United States; 2 College of Osteopathic Medicine Rocky Vista University Parker, CO United States; 3 Division of Dermatology Lehigh Valley Health Network Allentown, PA United States; 4 Department of Dermatology Duke University Hospital Durham, NC United States; 5 Dermatology Service US Department of Veterans Affairs Rocky Mountain Regional Medical Center Aurora, CO United States

**Keywords:** social media, TikTok, sponsorship, stewardship, ethics, dermatology, dermatologist, content analysis

The prevalence and nature of sponsored posts on Instagram by dermatologists have been well characterized within the literature [1]. Dermatologists use social media to provide education and recommend products; however, well-intended products and misinformation may be convoluted by financial or personal interests, potentially risking patient welfare. With the growing use of social media, we aimed to characterize the prevalence of sponsored posts by dermatologists on TikTok (ByteDance Ltd), a popular and rapidly growing video-sharing platform [2,3].

The following keywords were used to search for dermatologists’ profiles on TikTok: *dermatology*, *dermatologist*, *board-certified dermatologist*, and *doctor*. Exclusion criteria included profiles that were private or those without first or last name, degree, and specialty. Of the profiles included, the following data were collected for each profile: username, gender, training level (attending, resident, fellow), follower count (as a proxy for engagement), total number of posts, number of self-reported sponsored posts, number of dermatology-relevant sponsored posts, and category of sponsored posts (Table 1).

A total of 94 profiles were included; 67 (71.3%) belonged to female users and 27 (28.7%) belonged to male users. Attendings and residents/fellows accounted for 89.4% (n=84) and 10.6% (n=10), respectively. Of the 94 profiles, 38 (40.4%) had sponsored content, of which 32 (84.2%) were attending and 6 (15.8%) were trainee profiles. Among the 38 sponsored dermatologists, 34 (89.5%) had dermatology-relevant content. Residents/fellows had a greater median number of followers compared to attendings (28,650 vs 20,950). Sponsored dermatologists had a greater median number of followers than nonsponsored dermatologists (66,100 vs 1639) (Table 1). Sponsored dermatologists had significantly more followers even after subdividing by training level (*P*<.001 for attendings and *P*=.02 for residents/fellows). Table 2 displays the Spearman ρ and the corresponding *P* values for the number of followers, sponsored posts, and number of dermatologist sponsors.

Our study identified that less than half of the surveyed dermatologists on TikTok had sponsored posts. Dermatologists that had sponsored content had a higher number of followers, with a correlation between increased number of sponsored posts and number of followers. This highlights that product advertisement reaches larger audiences than medical education. Companies often seek highly followed “influencers” to maximize brand marketing [1,4].

Dermatologists with large followings and sponsored content have stewardship to educate their viewers and not only advertise. Dermatologists on TikTok can educate a wide audience about skin, nail, and hair health, and should do so mindfully. If patient welfare is prioritized above personal interests from sponsorship, dermatologists can have a profoundly positive impact on their TikTok audience. To prevent such biases, sponsored dermatologists must remain transparent on social media and disclose conflicts of interest clearly.

Social media is an integral part of society and influences the health care provider–patient dynamic, the dissemination of medical knowledge, and the delivery of care [5]. Dermatologists have leveraged TikTok to connect and educate the public and promote brands and best practices. The wide reach of social media augments the negative impact of promoting non–evidence-based products, sharing inaccurate information, and making wrongful claims. Dermatologists must be aware that the ethical standards that apply to patient care also apply to social media so that patient well-being is prioritized.

**Table 1:** Demographic characteristics.

Characteristic			Value^a^
**Sex, n (%)**			
	Female	67 (71.3)	
	Male	27 (28.7)	
**Training, n (%)**			
	Attendings	84 (89.4)	
	Residents/fellows	10 (10.6)	
**Sponsored dermatologists, n (%)**			38 (40.4)
	Attendings	32 (84.2)	
	Residents/fellows	6 (15.8)	
Dermatology sponsors, n (%)			34 (89.5)
Nondermatology sponsors, n (%)			4 (10.5)
Number of followers of attendings, median (IQR)			20,950 (703.8-148,600)
Number of followers of residents/fellows, median (IQR)			28,650 (1934.5-1,025,300)
Number of followers of sponsored attendings, median (IQR)			61,400 (18,150-409,425)
Number of followers of sponsored residents/fellows, median (IQR)			514,100 (35,847-4,550,000)
Number of followers of sponsored dermatologists, median (IQR)			66,100 (19,250-470,025)
Number of followers of nonsponsored dermatologists, median (IQR)			1639 (105.5-34,025)
Number of sponsored posts among dermatologists with sponsors, median (IQR)			8 (1.8-29.5)
Number of nonsponsored posts, median (IQR)			110 (41-232.3)
Number of posts with dermatology sponsors, median (IQR)			8 (1-28.8)
Number of posts with nondermatology sponsors, median (IQR)			0 (0-0)^b^
**Sponsored post details^c^**			
	Posts on skincare and hair, n	877^d^	
	Posts on cosmetics, n	6^d^	
	Posts on clothing/accessories, n	4^d^	
	Posts on prescription medications/procedures, n	1^d^	
	Miscellaneous posts, n	31^d^	

^a^Decimal places were rounded to the tenth decimal point when available.

^b^The median and IQR values were 0 with a maximum of 8. The mean was 0.6.

^c^Sponsored posts were subdivided into the following categories: skincare and hair, cosmetics, clothing/accessories, prescription medications/procedures, and miscellaneous.

^d^Values reported as total post count in each category.

**Table 2:** Demographic characteristics based on sponsorship status.

Characteristic	*P* value^a^	Correlation coefficient (Spearman *ρ*^b^)
Number of sponsored posts between male and female dermatologists	.07^c^	—^d^
Number of sponsored posts between attendings and residents/fellows	.84^e^	—
Number of followers between sponsored and unsponsored group	*<.001^f^*	—
Number of followers between attending sponsored and unsponsored group	*<.001^g^*	—
Number of followers between resident/fellow sponsored and unsponsored group	*.02^h^*	—
Number of followers and number of sponsored posts among dermatologists with sponsored posts	*<.001*	0.6216
Number of followers and number of dermatologist sponsors among all dermatologists with sponsored posts	*.001*	0.5136

^a^Italics denotes a significant difference.

^b^The Spearman correlation coefficient was used given the nonparametric nature of the data.

^c^Median (IQR): females, 4 (1-19); males, 24 (3-55).

^d^Not applicable.

^e^Median (IQR): attendings, 8 (2-39.5); residents, 13 (1-31.8).

^f^Median (IQR) for follower count in the sponsored group was 66,100 (19,250-470,025) while that of the nonsponsored group was 1639 (105.5-34,025).

^g^Median (IQR) for follower count in the attending sponsored group was 61,400 (18,150-409,425) while that of the attending nonsponsored group was 1443.5 (85.5-37,975).

^h^Median (IQR) for follower count in the resident/fellow sponsored group was 514,100 (35,847-4,550,000) while that of the resident/fellow nonsponsored group was 1853 (532.8-8679).
